# Supplementary material for: Uncertainties in deforestation emission baseline methodologies and implications for carbon markets
Source: Nat Commun. 2023 Dec 13;14:8277. doi: 10.1038/s41467-023-44127-9 (PMC10719246; doi:10.1038/s41467-023-44127-9)
Supplement: Supplementary file 1 — Supplementary Information [file 41467_2023_44127_MOESM1_ESM.pdf]

SUPPLEMENTARY INFORMATION

Uncertainties in deforestation emission baseline methodologies and implications for carbon markets

This PDF file includes:

Fig. S1-4

Note S1-3

Fig S1. Mean deforestation rates for each jurisdiction across all datasets and entire study period.

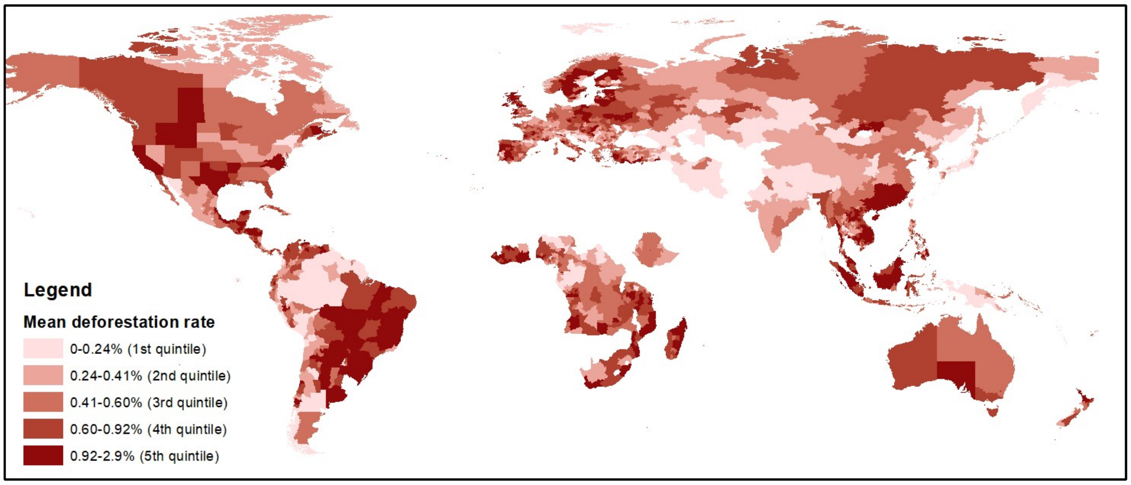

Fig S2. Mean forest cover for each jurisdiction across available years for: **(a)** European Space Agency Climate Change Initiative Land Cover (ESA CCI-LC) land cover 300 m, forest defined by land covers 50-90 and 160-170 where tree cover > 15% (Defourny et al., 2023), **(b)** Hansen Global Forest Change 30 m, forest defined by tree cover > 15% (Hansen et al., 2013), **(c)** Hansen Global Forest Change 30 m, forest defined by tree cover > 30% (Hansen et al., 2013), **(d)** Hansen Global Forest Change 30 m, forest defined by tree cover > 60% (Hansen et al., 2013), **(e)** MODIS MCD12Q1.061 land cover 500 m, forest defined by land covers 1-6 under classification Type 1 (International Geosphere Biosphere Programme classes) where tree cover > 60% (Friedl & Sulla-Menashe, 2015).

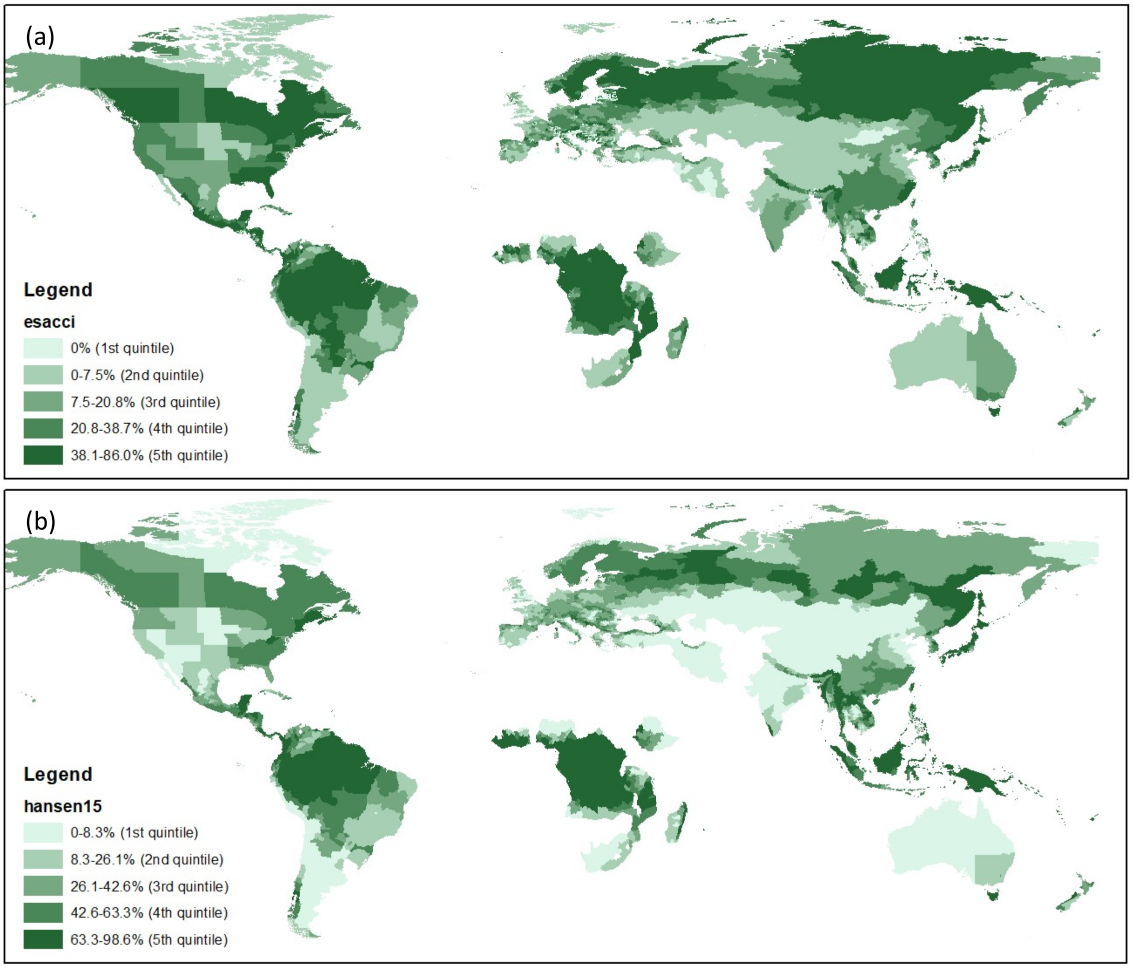

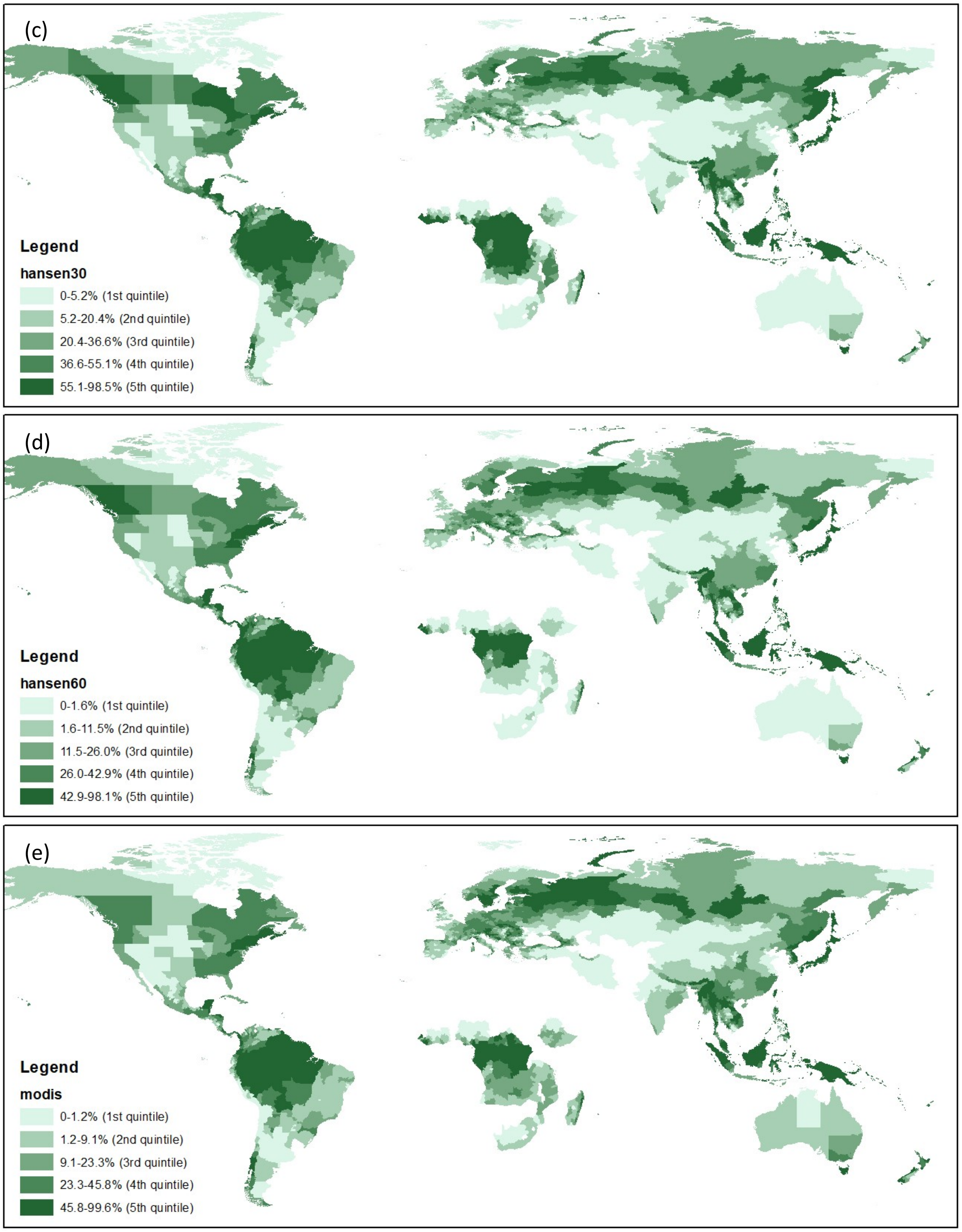
(c)
Legend
hansen30
0-5.2% (1st quintile)
5.2-20.4% (2nd quintile)
20.4-36.6% (3rd quintile)
36.6-55.1% (4th quintile)
55.1-98.5% (5th quintile)
(d)
Legend
hansen60
0-1.6% (1st quintile)
1.6-11.5% (2nd quintile)
11.5-26.0% (3rd quintile)
26.0-42.9% (4th quintile)
42.9-98.1% (5th quintile)
(e)
Legend
modis
0-1.2% (1st quintile)
1.2-9.1% (2nd quintile)
9.1-23.3% (3rd quintile)
23.3-45.8% (4th quintile)
45.8-99.6% (5th quintile)

Fig S3. (a) Forest area (log-10 adjusted) against % variability (CV) for each of n = 2,794 jurisdictions.

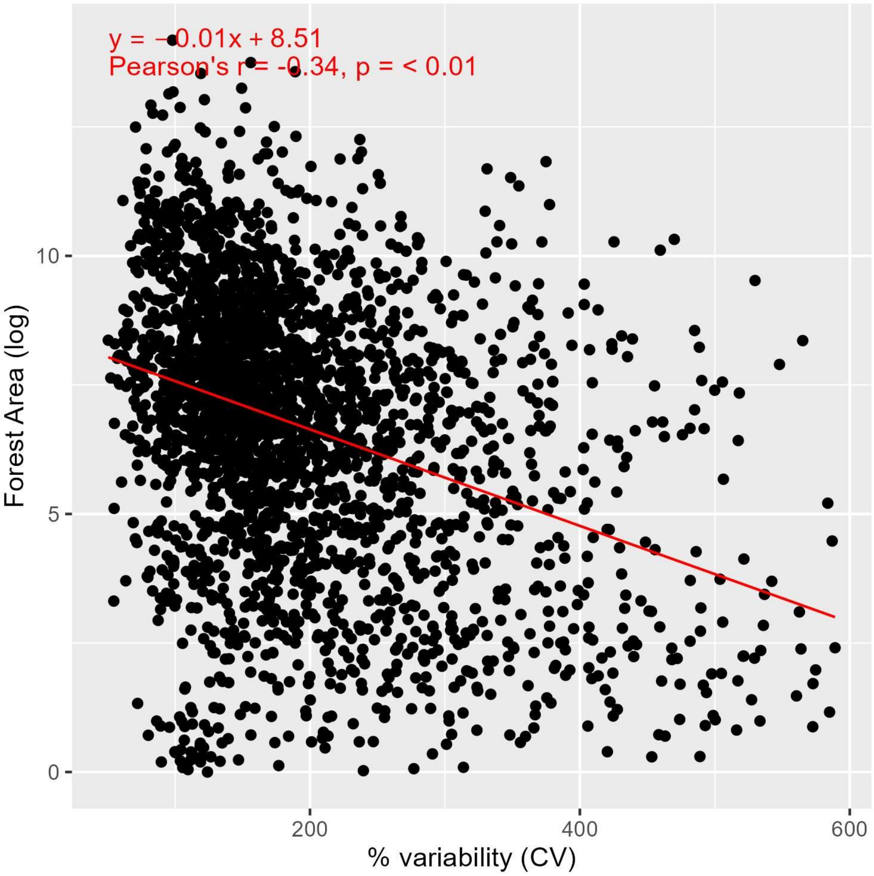

Fig S3. (b) Forest area (log-10 adjusted) against forecast error for each of *n* = 2,794 jurisdictions.

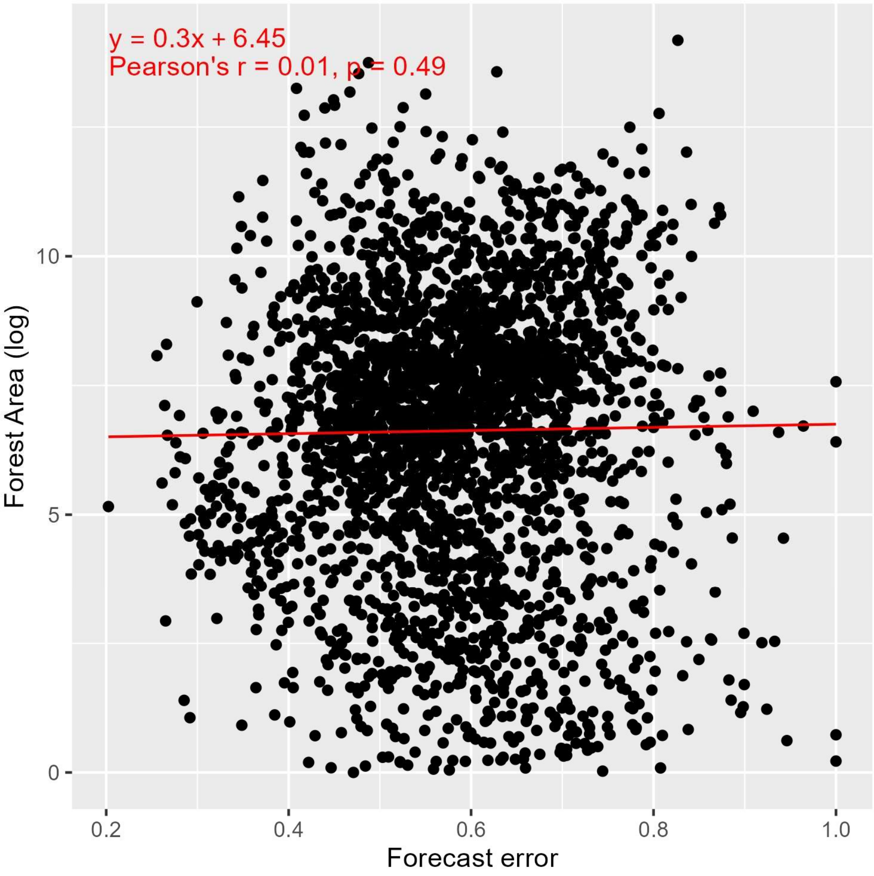

Fig S3. (c) Forest area (log-10 adjusted) against % uncertainty (90% CI) for each of *n* = 2,794 jurisdictions.

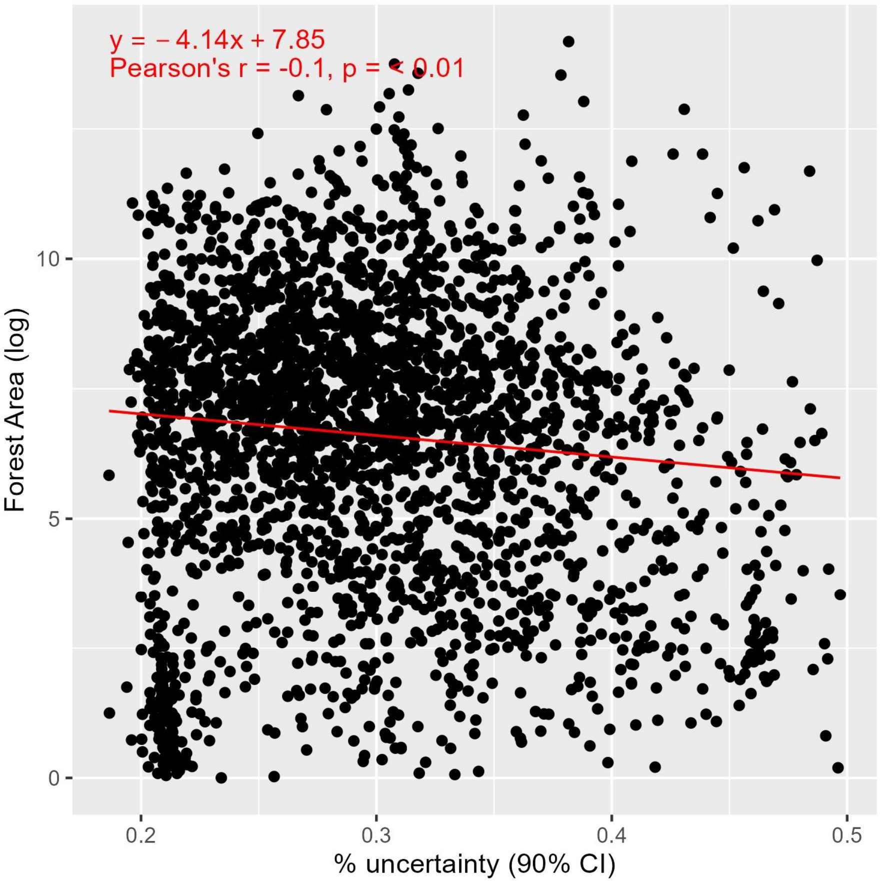

Fig S4. Global map of jurisdictional forecast error of deforestation emission baselines (each point represents one jurisdiction) for different projection approaches: (**a**) median of all deforestation projection approaches, (**b**) historical average, (**c**) linear time function, (**d**) 2nd order polynomial time function, (**e**) global linear modelling (all 11 driver variables), (**f**) mean of 300 repetitions of global linear model (3-10 random combinations of driver variables), (**g**) regional linear modelling (all 11 driver variables), (**f**) mean of 300 repetitions of regional linear models (3-10 random combinations of driver variables).

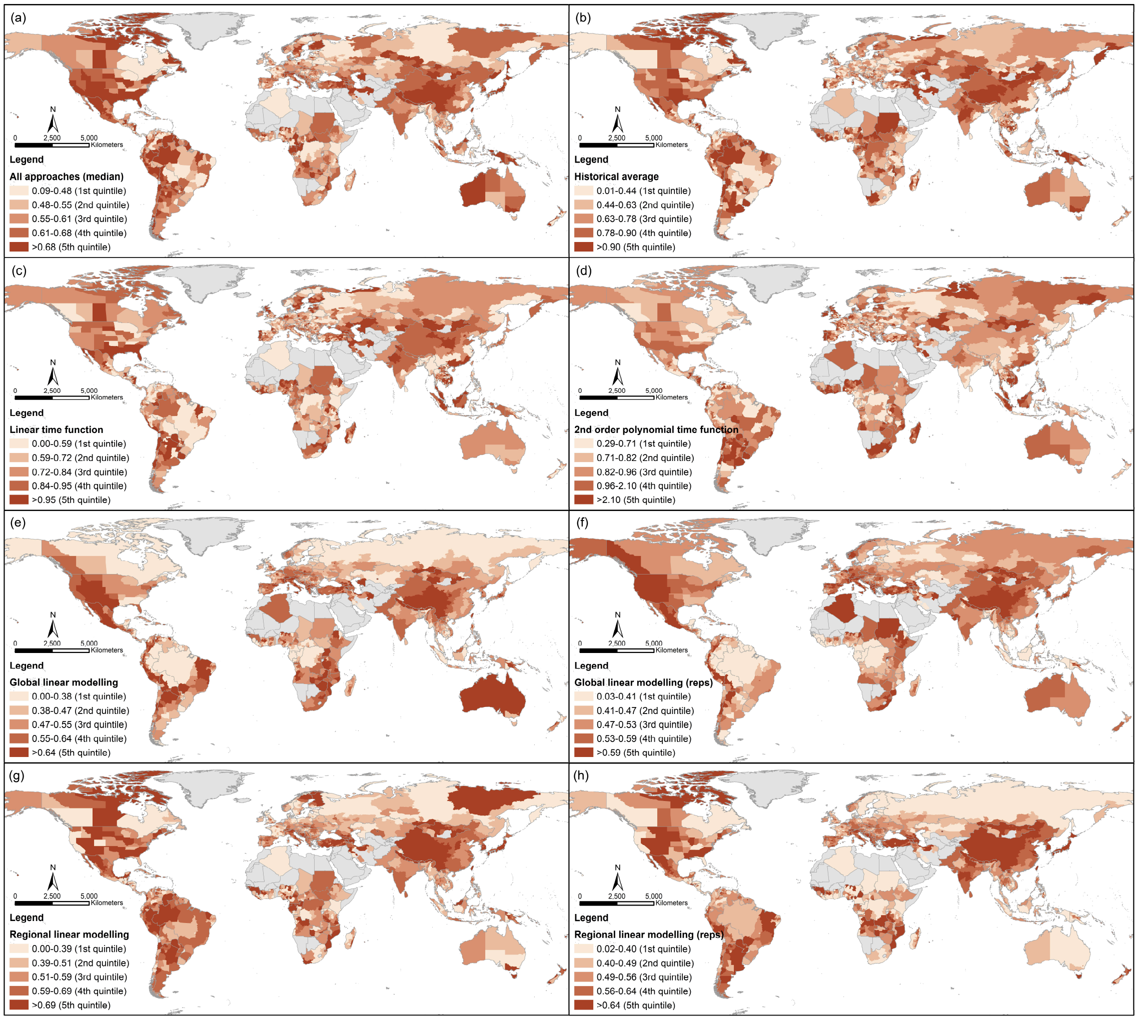

Note S1. General guidelines and recommendations from carbon standards on baseline estimation and deforestation risk modelling.

1. Number of historical reference years for country FRL/FREL submissions
   - Forest Carbon Partnership Facility - Methodological Framework Criterion 11
     i. Around 10 years
     ii. No more than 15 years
   - Green Climate Fund
     i. Preference for 10-15 year period (As cited in Nigeria FRL submission)
     ii. Allows for reference period of 5 to 20 years (As cited in Mozambique FRL submission)

2. Number of historical reference years for deforestation risk mapping
   - VCS VM0015
     i. Start date of reference period to not exceed 10-15 years in the past
     ii. End date to be as close as possible to the project start date

3. Carbon pools for FRL/FREL submissions
   - IPCC provides default values for dead wood, litter and soil organic carbon (As cited in Belize FRL submission, but unable to find the given values)

4. Tools and methods for deforestation risk mapping

   Any models and software can be selected, as long as they are peer-reviewed and prove to conform to the methodology

   - Examples of software named by VCS:
     i. Geomod
     ii. IDRISI
     iii. Dinamica-EGO
     iv. Clue
     v. Land-use Change Modeller
   - As recommended in VCS VM0015:
     i. *"Several model/software are available and can be used to perform these tasks in slightly different ways, such as Geomod, Idrisi Taiga, Dinamica Ego, Clue, and Land-Use Change Modeler. The model/software used must be peer-reviewed and must be consistent with the methodology (to be proven at validation)."*
     ii. *"Models use different techniques to produce Risk Maps and algorithms may vary among the different modeling tools. Algorithms of internationally peer-reviewed modeling tools are eligible to prepare deforestation risk maps, provided they are shown to conform to the methodology at time of validation."*

Note S2. Variables in ex-ante estimation of emission reductions.

Selection of variables tend to differ between countries due to national circumstances that affect availability of data.

Variables used in FRL/FREL constructions are similar, but it is unclear whether there are specific recommendations for all variables. The following variables and recommendations are based on the VCS VM0015 methodology:

1. Carbon pools
   * Significant = constitutes >5% of total GHG benefits generated
   - AGB (tree) is the only mandatory carbon pool across all projects
   - Harvested wood products must be included when significant
   - The following are only mandatory when requirements are met for specific projects:
     i. AGB (non-tree)
     ii. Dead wood
     iii. Litter
   - The following are recommended and not mandatory
     i. BGB
     ii. Soil organic carbon

2. GHG sources
   - No mandatory sources, significance to be determined for specific projects

3. Forest classification
   - Minimum classes are “forest” and “non-forest”
   - Forest strata dependent on resolution of national data

4. Reference years
   - Long reference periods could result in the inclusion of historical patterns that do not reflect predicted future patterns
   - Short reference periods may be insufficient in capturing the true historical trend of emissions
   - For FRL/FREL: Availability of reliable national Activity Data (AD) and Emission Factors (EF), satellite imagery, occurrence of natural disasters (e.g. hurricane Maria affecting Dominica’s FRL calculation)

Note S3. Brief explanation of rationale and theoretical background for variables used in linear modelling.

1. Elevation and slope

   Elevation is an important driver for biogeographical patterns and thus ecological processes and zones; human settlement dynamics and government policies in certain countries may also follow elevational zones (Bhattarai et al., 2009; Trigueiro et al., 2020).

   Steep slopes may inhibit deforestation as these are unconducive for agriculture and may be less accessible (Carvalho Lima et al., 2018; Trigueiro et al., 2020).

2. Temperature and precipitation

   Temperature and precipitation are climatic drivers which can affect fire dynamics in areas which are fire-prone, such as the ignition and ease of spread; hotter and drier climatic conditions may thus increase deforestation risk (Aragão et al., 2008; Laurance et al., 2002). Certain climates more conducive for agriculture may be attractive for agriculture-driven deforestation (Bax & Francesconi, 2018; Grau et al., 2005).

3. Gross Domestic Product (GDP) and Human Development Index

   The environmental Kuznets curve suggests that a metric for environmental degradation (e.g. deforestation) rises at first with rising development due to greater demand for resources and consumption, but environmental degradation drops after a turning point as development brings technological improvements and greater demand for environmental amenities. Different relationships between GDP/development and deforestation have been suggested (Koop & Tole, 1999).

4. Nightlight intensity and population density

   Settlement patterns, reflected in population density, may influence deforestation drivers such as agricultural expansion, infrastructure development, and resource extraction (Teo et al., 2019; Tritsch & Le Tourneau, 2016). This may come in the form of large-scale agriculture, export-driven agriculture, or subsistence agriculture and the extraction of firewood for domestic use (Fisher, 2010).

   Nightlight intensity is frequently used as a proxy for population density and economic activity, and to complement such datasets (Dorji et al., 2019; Liu et al., 2021).

5. Percentage forest area and percentage agricultural area

   The percentage of remaining forest area, and percentage of agricultural area, are key factors in forest transition theory (Mather & Needle, 1992). Forest transition theory describes the relation between the stages in development (reflected by agricultural land) and forest cover.

6. Percentage land area occupied by mining land uses

   Mining is a key driver for deforestation in many areas (Alvarez-Berrios & Mitchell Aide, 2015; Ang et al., 2021; Ranjan, 2019). Deforestation pathways may include infrastructure development, urban expansion for workers' housing and services, as well as broader supply chains (Sonter et al., 2017).

7. Percentage land area occupied by tree plantations
   Tree plantations, such as oil palm, are key drivers for deforestation in many areas (Carlson et al., 2013; Koh & Wilcove, 2008).

Alvarez-Berrios, N. L., & Mitchell Aide, T. (2015). Global demand for gold is another threat for tropical forests. *Environmental Research Letters*. https://doi.org/10.1088/1748-9326/10/1/014006

Ang, M. L. E., Arts, D., Crawford, D., Labatos, B. V., Ngo, K. D., Owen, J. R., Gibbins, C., & Lechner, A. M. (2021). Socio-environmental land cover time-series analysis of mining landscapes using Google Earth Engine and web-based mapping. *Remote Sensing Applications: Society and Environment*, 21, 100458. https://doi.org/10.1016/J.RSASE.2020.100458

Aragão, L. E. O. C., Malhi, Y., Barbier, N., Lima, A., Shimabukuro, Y., Anderson, L., & Saatchi, S. (2008). Interactions between rainfall, deforestation and fires during recent years in the Brazilian Amazonia. *Philosophical Transactions of the Royal Society B: Biological Sciences*, 363(1498), 1779–1785. https://doi.org/10.1098/RSTB.2007.0026

Bax, V., & Francesconi, W. (2018). Environmental predictors of forest change: An analysis of natural predisposition to deforestation in the tropical Andes region, Peru. *Applied Geography*, 91, 99–110. https://doi.org/10.1016/J.APGEOG.2018.01.002

Bhattarai, K., Conway, D., & Yousef, M. (2009). Determinants of deforestation in Nepal's Central Development Region. Journal of Environmental Management, 91(2), 471–488. https://doi.org/10.1016/J.JENVMAN.2009.09.016

Carlson, K. M., Curran, L. M., Asner, G. P., Pittman, A. M. D., Trigg, S. N., & Marion Adeney, J. (2013). Carbon emissions from forest conversion by Kalimantan oil palm plantations. *Nature Climate Change*. https://doi.org/10.1038/nclimate1702

Carvalho Lima, T., Carvalho Ribeiro, S., & Soares-Filho, B. (2018). Integrating Econometric and Spatially Explicit Dynamic Models to Simulate Land Use Transitions in the Cerrado Biome. 399–417. https://doi.org/10.1007/978-3-319-60801-3_19

Dorji, U., Siripanpornchana, C., Surasvadi, N., Plangprasopchok, A., & Thajchayapong, S. (2019). Exploring Night Light as Proxy for Poverty and Income Inequality Approximation in Thailand. *IEEE Region 10 Annual International Conference*, Proceedings/TENCON, 2019-October, 1082–1087. https://doi.org/10.1109/TENCON.2019.8929247

Fisher, B. (2010). African exception to drivers of deforestation. *Nature Geoscience* 2010 3:6, 3(6), 375–376. https://doi.org/10.1038/NGEO873

Grau, R. H., Gasparri, N. I., & Aide, M. T. (2005). Agriculture expansion and deforestation in seasonally dry forests of north-west Argentina. *Environmental Conservation*, 32(2), 140–148.

Koh, L. P., & Wilcove, D. S. (2008). Is oil palm agriculture really destroying tropical biodiversity? *Conservation Letters*, 1(2), 60–64. https://doi.org/10.1111/j.1755-263x.2008.00011.x

Koop, G., & Tole, L. (1999). Is there an environmental Kuznets curve for deforestation? *Journal of Development Economics*, 58(1), 231–244. https://doi.org/10.1016/S0304-3878(98)00110-2

Laurance, W. F., Albernaz, A. K. M., Schroth, G., Fearnside, P. M., Bergen, S., Venticinque, E. M., & Da Costa, C. (2002). Predictors of Deforestation in the Brazilian Amazon. *Journal of Biogeography*, 28(5/6), 737–748. https://www.jstor.org/stable/827480

Liu, H., He, X., Bai, Y., Liu, X., Wu, Y., Zhao, Y., & Yang, H. (2021). Nightlight as a Proxy of Economic Indicators: Fine-Grained GDP Inference around Chinese Mainland via Attention-Augmented CNN from Daytime Satellite Imagery. *Remote Sensing* 2021, 13(11), 2067. https://doi.org/10.3390/RS13112067

Mather, A. S., & Needle, C. L. (1992). The Forest Transition. *Area*, 24(4), 367–379. https://www.jstor.org/stable/20003181

Ranjan, R. (2019). Assessing the impact of mining on deforestation in India. *Resources Policy*, 60, 23–35. https://doi.org/10.1016/j.resourpol.2018.11.022

Sonter, L. J., Herrera, D., Barrett, D. J., Galford, G. L., Moran, C. J., & Soares-Filho, B. S. (2017). Mining drives extensive deforestation in the Brazilian Amazon. *Nature Communications*, 8(1), 1–7. https://doi.org/10.1038/s41467-017-00557-w

Teo, H. C., Lechner, A. M., Walton, G. W., Chan, F. K. S., Cheshmehzangi, A., Tan-mullins, M., Chan, H. K., Sternberg, T., & Campos-arceiz, A. (2019). Environmental Impacts of Infrastructure Development under the Belt and Road Initiative. *Environments*, 6(72).

Trigueiro, W. R., Nabout, J. C., & Tessarolo, G. (2020). Uncovering the spatial variability of recent deforestation drivers in the Brazilian Cerrado. *Journal of Environmental Management*, 275, 111243. https://doi.org/10.1016/J.JENVMAN.2020.111243

Tritsch, I., & Le Tourneau, F. M. (2016). Population densities and deforestation in the Brazilian Amazon: New insights on the current human settlement patterns. *Applied Geography*, 76, 163–172. https://doi.org/10.1016/J.APGEOG.2016.09.022
