# Supplementary material for: Uncertainties in deforestation emission baseline methodologies and implications for carbon markets
Source: Nat Commun. 2023 Dec 13;14:8277. doi: 10.1038/s41467-023-44127-9 (PMC10719246; doi:10.1038/s41467-023-44127-9)
Supplement: Supplementary file 3 — Description of Additional Supplementary Files [file 41467_2023_44127_MOESM3_ESM.pdf]

Description of Additional Supplementary Files

File Name: Supplementary Data 1
Description: This file contains a summary of project details, methodology variables, and deforestation risk mapping variables and methods for VCS VM0015 Projects, Plan Vivo Projects, and UNFCCC FRL/FREL Submissions.
